# Exploring participant’s experiences in a multifamily therapy group on schizophrenia : a qualitative approach

**DOI:** 10.1192/j.eurpsy.2024.1491

**Published:** 2024-08-27

**Authors:** O. Amiot, C. Genis, L. Champlon, S. Said, L. Baziret

**Affiliations:** 92100, GH Paul Guiraud, Boulogne Billancourt, France

## Abstract

**Introduction:**

Prevention of relapse in schizophrenia is a major public health issue. A recent network meta-analysis investigating interventions for relapse prevention in schizophrenia found that the efficacity of family psychoeducation and systemic integrated interventions were superior to treatment as usual at 12 months (Bighelli I, Leucht S et al. Lancet Psychiatry 2021). Other studies also found that multi-family therapies (MFT) were superior to treatment as usual and family psychoeducation in preventing relapses at 2 and 4 years (McFarlane WR, Lukens EP et al. Archives of General Psychiatry.1995). Considering this, we developped in our community center an MFT program based on systemic approach and psychoeducation.

**Objectives:**

Investigate the subjective experience of participants of an MFT group focusing on schizophrenia.

**Methods:**

A qualitative study was designed to explore personal experience of participants using the Interpretative Phenomenological Analysis (IPA) method in order to analyse participant’s feedback during semi-directive interviews. By using IPA, participants are experts of their experience. Eight participants took part in this study: 4 patients and 4 parents.

**Results:**

Within all participant’s feedback around 10 different themes emerged. We identified three major themes which we have described as: “Affiliation to the group”, “Framework of Discovery”, “Benefits of MFT”.

According to “Affiliation to the group”, all participants report movements of adhesion or rejection towards the group. This theme has been subdivided into two sub-themes: “Temporality”, and “Identification/differentiation”. These sub-themes revealed inter-individuals’ differences.

According to “Framework of discovery”, the MFT group has been identified as a secure place allowing self and other’s discovery place. This theme has been divided into two sub-themes: “discovering skills” and “improving oneself and relatives’ understanding”. Participants experience taking a step back and decentering oneself from usual personnal position.

According to “Benefits of MFT”, participants report the feeling of belonging to a group, the impact on self-esteem, on mentalization skills, and on the reflexion on family members’ experience of the disease.

**Conclusions:**

This study is, to our knowledge, the first qualitative study examining the subjective experience of members who participated in a psychoeducational and systemic MFT group focusing on schizophrenia. It provides insight into the families’ experience, both from the patient’s and from each family member’s perspective. Results highlight that participants seized the MFT group as a learning space at several levels: personal, intra-family and inter-family.

These data could enlighten professionals working with families on the potential apprehensions of participants, their representations of the group and what process MFT could initiate.

**Disclosure of Interest:**

None Declared

